# Supplementary material for: Dual-Specificity Protein Phosphatase 4 (DUSP4) Overexpression Improves Learning Behavior Selectively in Female 5xFAD Mice, and Reduces β-Amyloid Load in Males and Females
Source: Cells. 2022 Dec 1;11(23):3880. doi: 10.3390/cells11233880 (PMC9737364; doi:10.3390/cells11233880)
Supplement: Supplementary file 1 [file cells-11-03880-s001.zip › cells-1885836-supplementary.pdf]

SUPPLEMENTARY MATERIALS

Supplementary Figures

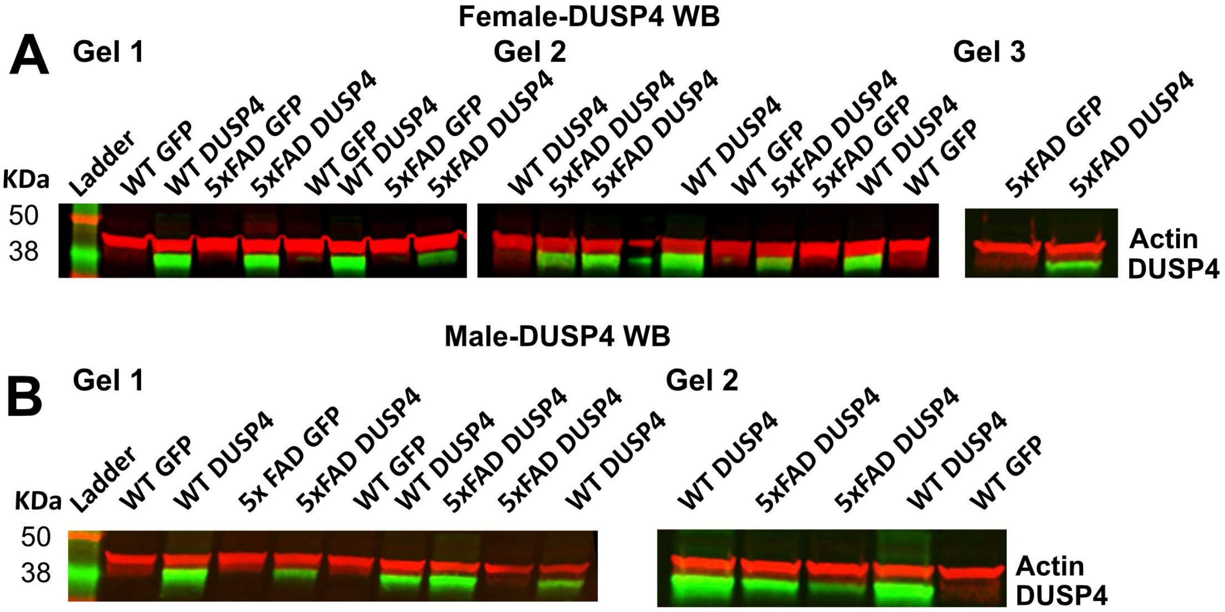

Figure S1. Western blot analyses of DUSP4 protein expression for both female and male 5xFAD or WT mice overexpressing DUSP4. (A) Hippocampal DUSP4 protein level from female mice 5xFAD or WT overexpressing DUSP4, one month after the injection of AAV5-DUSP4. (B) Hippocampal DUSP4 protein level from male 5xFAD or WT mice overexpressing DUSP4, one month after the injection of AAV5-DUSP4. The quantification data is shown is in Fig. 2D right panel.

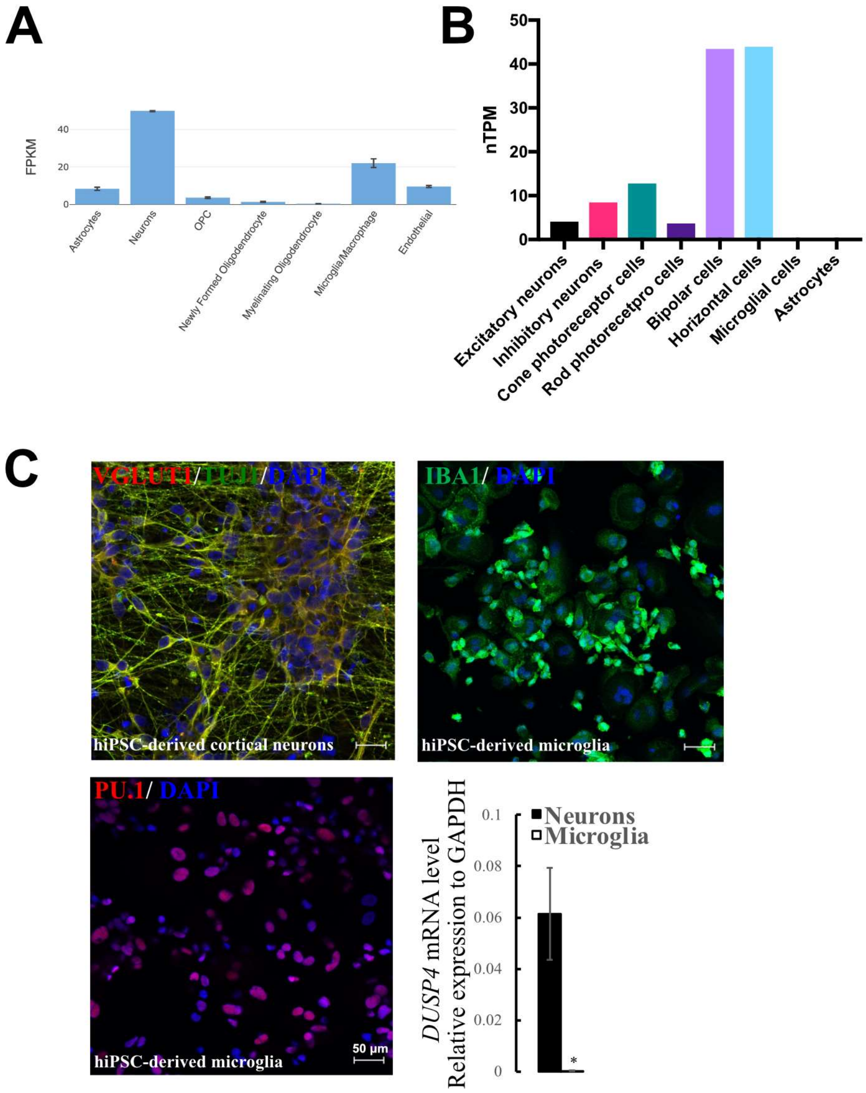

Figure S2. Neuronal cells have higher level of *Dusp4* expression. (A) Cell-type expression of mouse *Dusp4* mRNA, source www.brainrnaseq.org. (B) Single cell RNAseq data of human *DUSP4* expression, source www.proteinatlas.org. (C) Representative image of VGLUT1^{+}/TUJ1^{+} hiPSC-derived cortical neurons expressing VGLUT1 (top left) and hiPSC-derived microglial cells expressing IBA1 and PU.1 (top right and bottom left, respectively), and qPCR expression analysis of *DUSP4* in hiPSC-derived cells, cortical neurons and microglial cells (bottom right). VGLUT1, Vesicular glutamate transporter 1; TUJ1, beta-tubulin III; IBA1, Ionized calcium binding adaptor molecule 1.

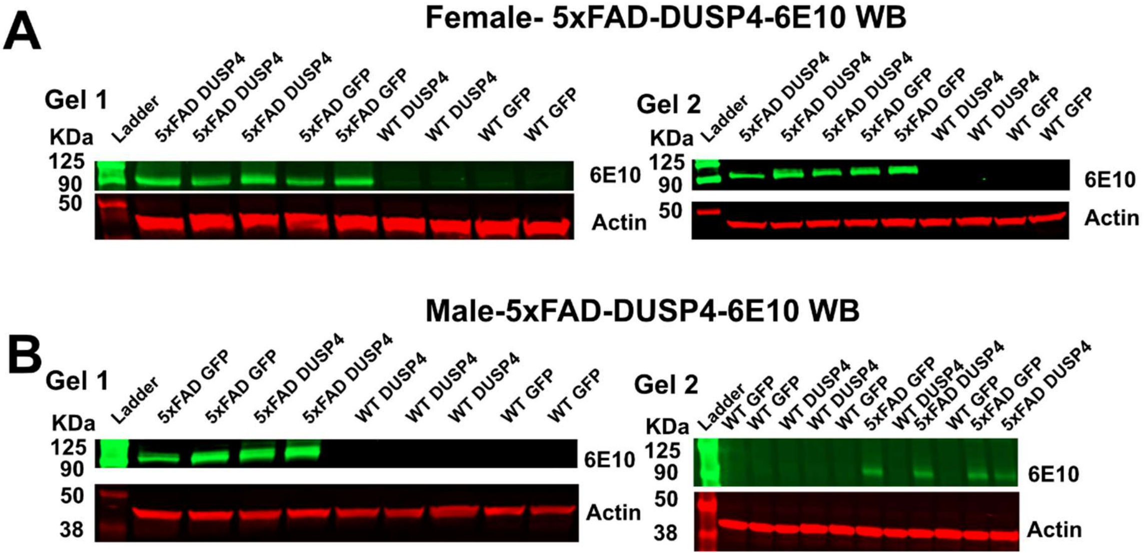

Figure S3. Western blot analyses of human APP (clone 6E10) protein levels for female and male mice overexpressing DUSP4. Human APP protein levels in hippocampal RIPA lysates of (A) female and (B) male 5xFAD or WT mice overexpressing DUSP4. The quantification data is shown in Fig. 4C.

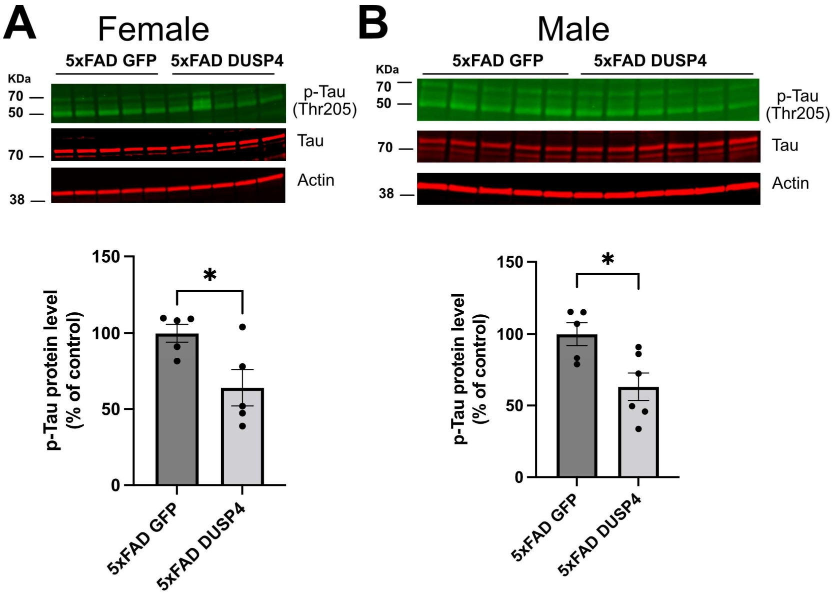

Figure S4. DUSP4 overexpression decreases phospho-Tau levels. Western blot analysis of protein RIPA extracts from hippocampi showed a significant decrease of p-Tau (Thr205) levels in DUSP4-overexpressing (A) female and (B) male 5xFAD mice, n = 4-5 mice/group. Statistical analysis was performed using a Student's t-test, $*p<0.05$.

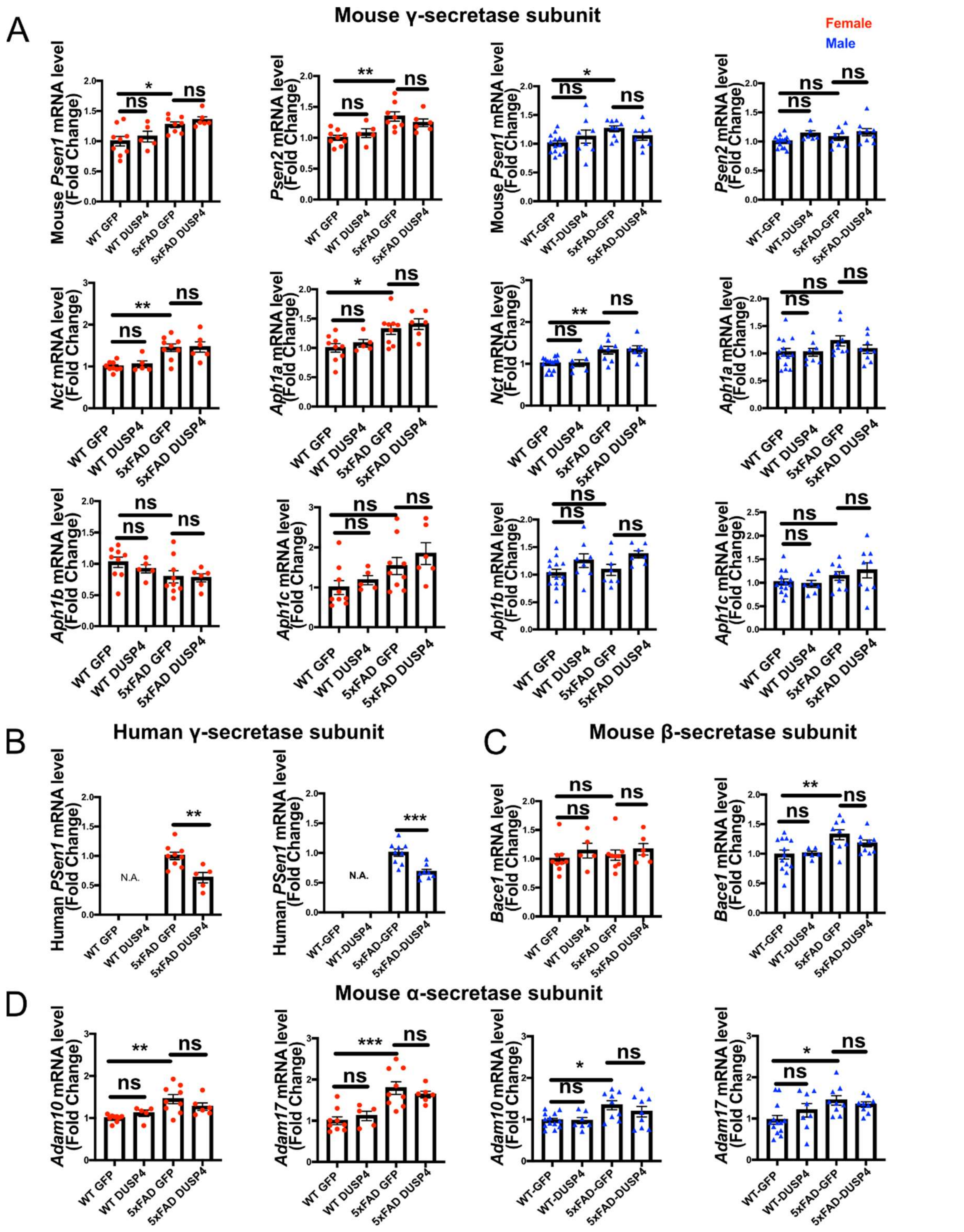

Figure S5. DUSP4 overexpression did not alter mouse APP processing enzyme mRNA levels, but reduced human *PSEN1* mRNA levels. (A) Hippocampal mouse *Ps1*, *Ps2, Nct, Aph1a,* and *Aph1c* mRNAs were assayed for female and male 5xFAD and WT mice overexpressing DUSP4 or GFP, n = 5-14 mice per group. (B) Female and male 5xFAD and WT overexpressing DUSP4 or GFP were assayed for human *PSEN1* mRNA in hippocampus by RT-PCR, n=5-9 mice per group. (C) Female and male 5xFAD and WT overexpressing DUSP4 or GFP were assayed for BACE1

mRNA in hippocampus by RT-PCR. n = 5-14 mice per group. (D) Female and male 5xFAD and WT overexpressing DUSP4 or GFP were assayed for mouse *Adam10*, and mouse *Adam17* mRNAs in hippocampus by RT-PCR. n = 5-14 mice per group. Error bars represent means ± SEM. Statistical analyses were performed using a Student's t-test for human *PSEN1* mRNA or One-Way ANOVA followed by a Tukey's post-hoc test for mouse *Psen1*, *Psen2, Bace1, Nct*, *Aph1a*, *Aph1b*, and *Aph1c* mRNAs, *p<0.05, **p<0.01, ***p< 0.001, N.A. = not applicable, ns = nonsignificant.

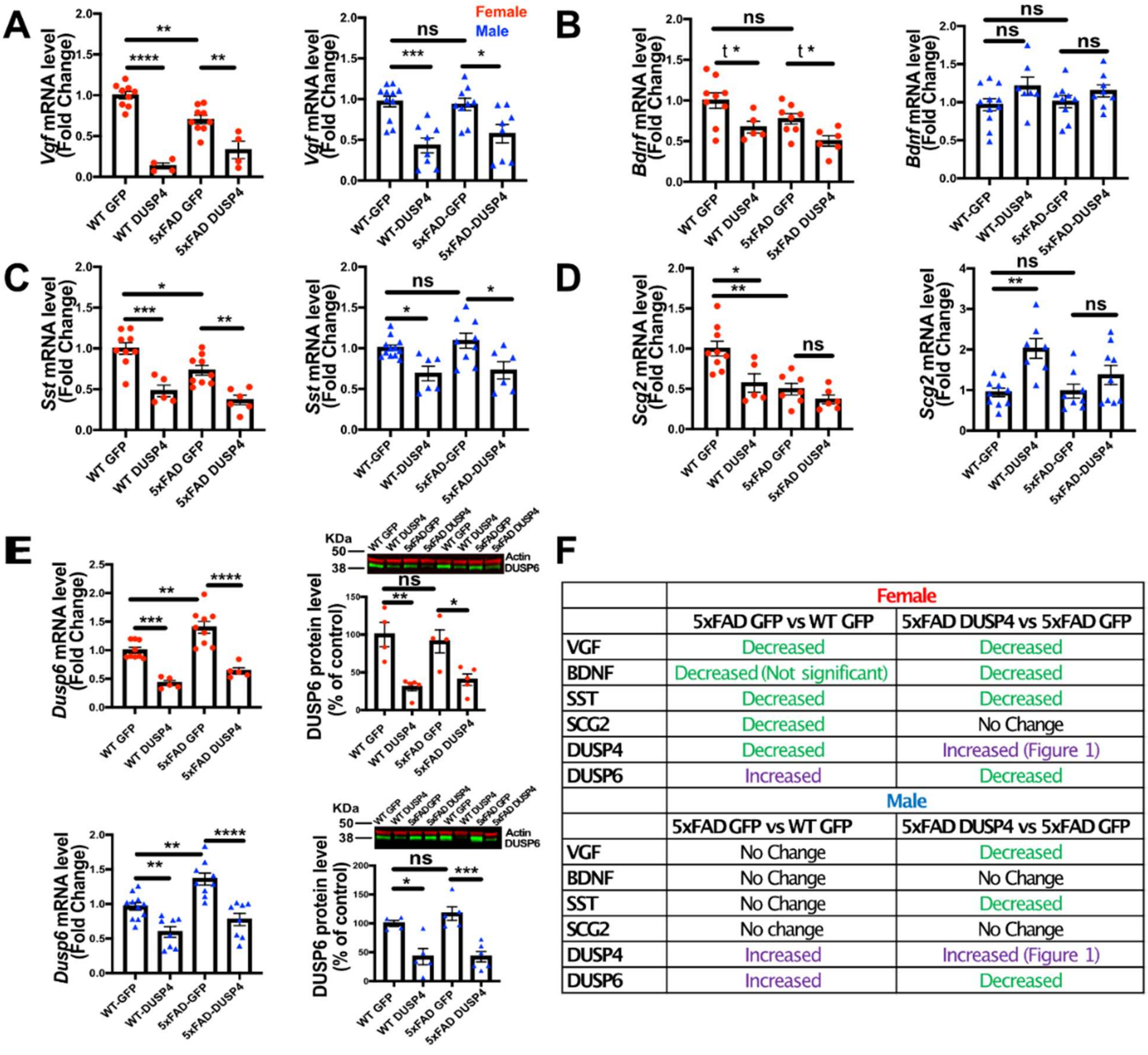

	Female	
	5xFAD GFP vs WT GFP	5xFAD DUSP4 vs 5xFAD GFP
VGF	Decreased	Decreased
BDNF	Decreased (Not significant)	Decreased
SST	Decreased	Decreased
SCG2	Decreased	No Change
DUSP4	Decreased	Increased (Figure 1)
DUSP6	Increased	Decreased
	Male	
	5xFAD GFP vs WT GFP	5xFAD DUSP4 vs 5xFAD GFP
VGF	No Change	Decreased
BDNF	No Change	No Change
SST	No Change	Decreased
SCG2	No change	No Change
DUSP4	Increased	Increased (Figure 1)
DUSP6	Increased	Decreased

Figure S6. The effects of DUSP4 on VGF network-associated genes in 5xFAD and WT mice. (A) Hippocampal *Vgf* mRNA level in 5xFAD or WT mice overexpressing DUSP4 compared to control (WT-GFP), n = 4-10 mice per group. (B) Hippocampal *Bdnf* mRNA level in 5xFAD or WT mice overexpressing DUSP4 compared to the control, n = 5-10 mice per group. (C) Hippocampal *Sst* mRNA level in 5xFAD or WT mice overexpressing DUSP4 compared to the control, n = 5-10 mice per group. (D) Hippocampal *Scg2* mRNA level in 5xFAD or WT mice overexpressing DUSP4 compared to control, n = 5-11 mice per group. (E) Hippocampal *Dusp6* mRNA and protein

levels in 5xFAD or WT mice overexpressing DUSP4 compared to control, n = 4-11 mice per group. (F) Summary table of changes in VGF network-associated gene expression as a result of DUSP4 overexpression. Statistical analysis was performed using a one-way ANOVA followed by a Tukey's post-hoc test for all graphs, except when t* is noted for a student's t-test comparison that was carried out to follow up a trend in a nonsignificant result, *p<0.05, **p<0.01, ***p< 0.001, ****p<0.0001; ns nonsignificant.

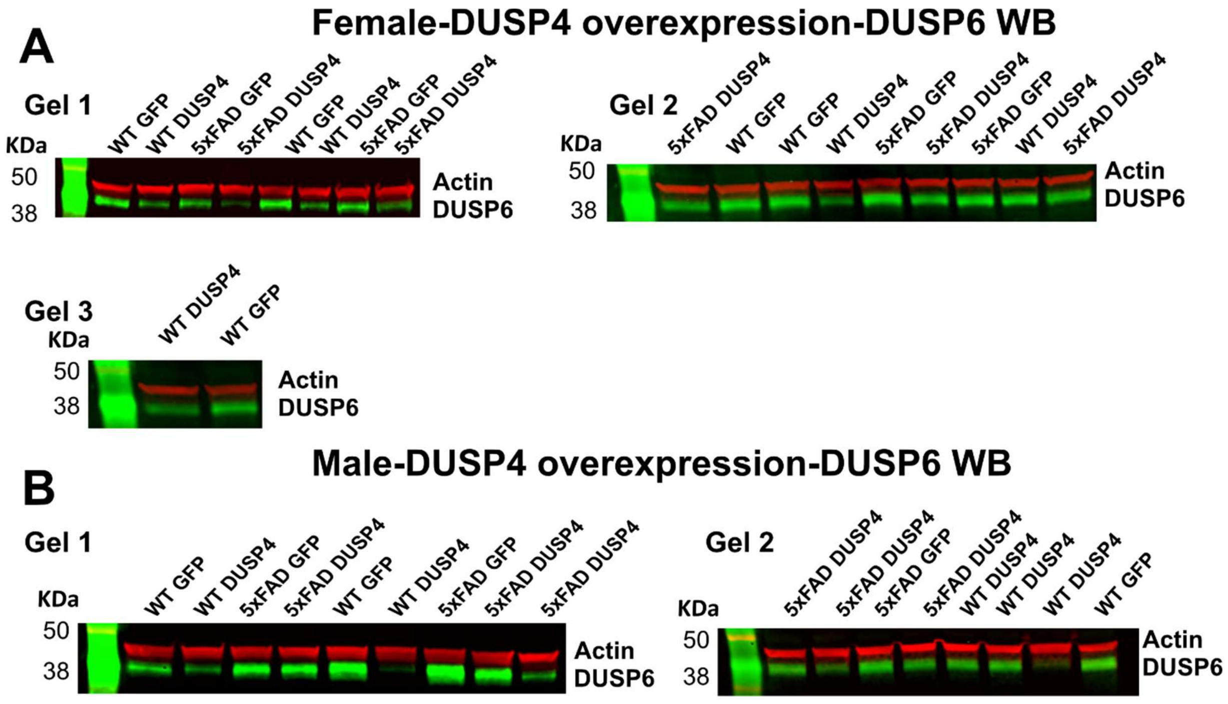

Figure S7. Western blot analyses of DUSP6 protein expression for both female and male 5xFAD or WT mice overexpressing DUSP4. (A) Hippocampal DUSP6 protein level from female 5xFAD or WT mice overexpressing DUSP4. (B) Hippocampal DUSP6 protein level from male 5xFAD or WT mice overexpressing DUSP4. The quantification data is shown in Fig. 5E right top and bottom panel.

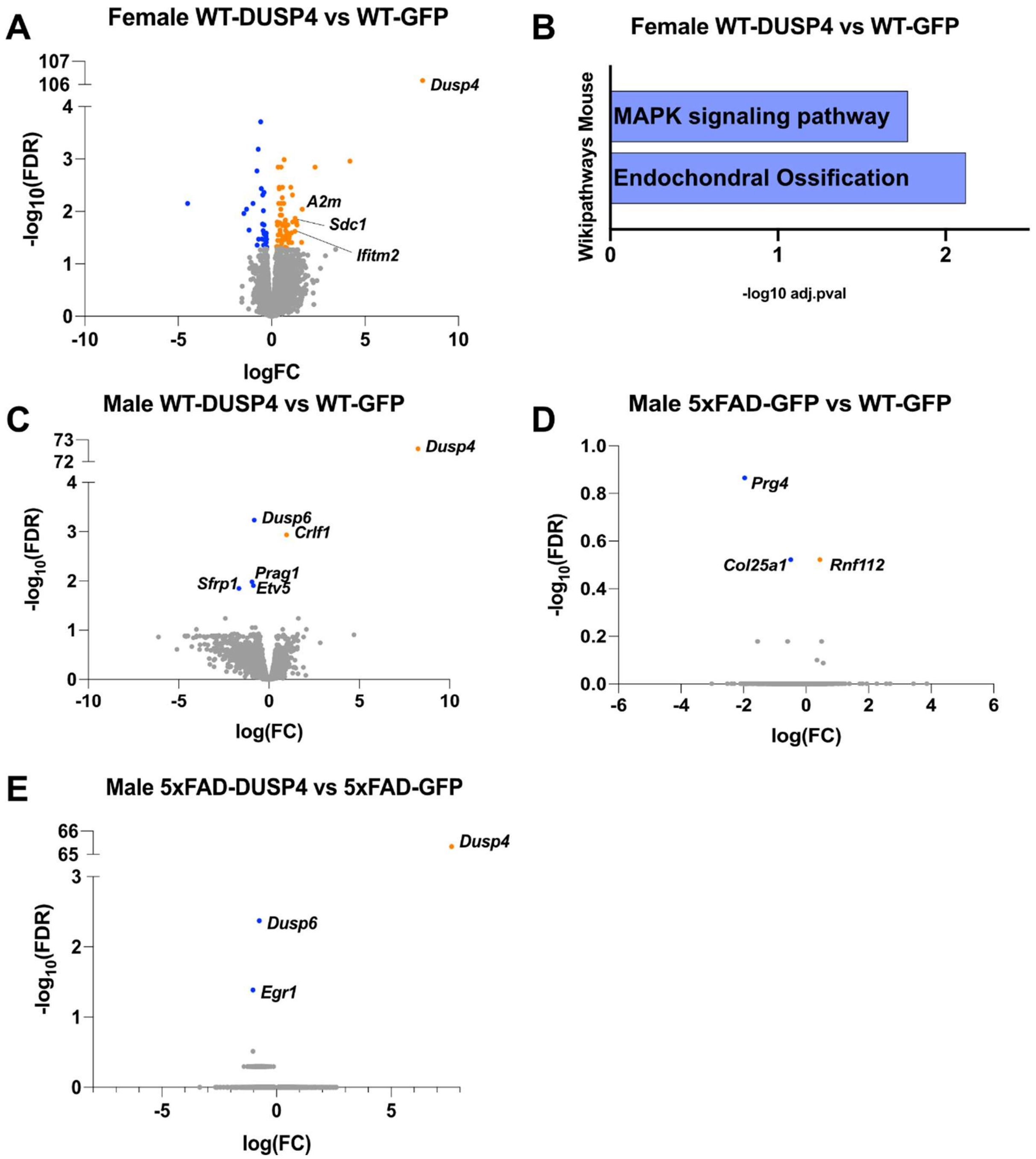

Figure S8. RNAseq analyses of hippocampi from female and male 5xFAD and WT overexpressing DUSP4 or GFP. (A) Volcano plot representation of female 5xFAD-DUSP4 compared to WT-GFP. (B) Pathway enrichment analysis of the DEGs by Enrichr. (C) Volcano plot representation of male WT-DUSP4 vs WT-GFP. (D) Volcano plot representation of male 5xFAD-GFP vs WT-GFP. (E) Volcano plot representation of male 5xFAD-DUSP4 vs 5xFAD-GFP. Orange dots represent upregulated DEGs and blue dots represent downregulated DEGs, FDR < 0.05.

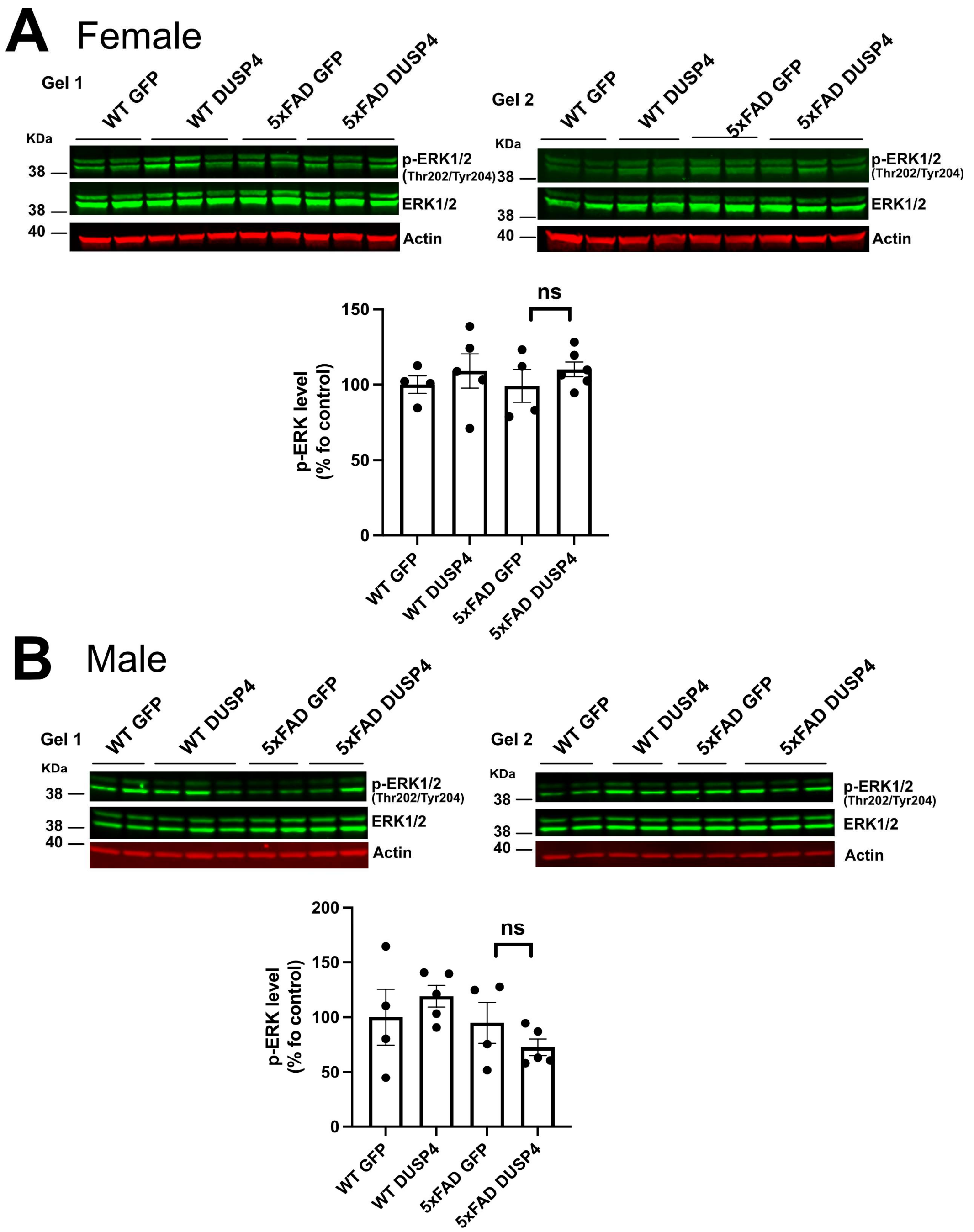

Figure S9. DUSP4 overexpression does not change p-ERK levels in either female or male 5xFAD mice. (A) Western blot analysis of female protein extracts from dorsal hippocampi showed no significant of change p-ERK levels in DUSP4-overexpressing female 5xFAD compared to GFP-overexpressing female 5xFAD mice, n = 4-6 mice/group. (B) Western blot analysis of male protein from dorsal hippocampi showed no significant change of p-ERK levels in DUSP4-overexpressing male 5xFAD compared to GFP-overexpressing male 5xFAD mice, n = 4-5 mice/group. Statistical analysis was performed using a one-way ANOVA followed by a Tukey's post-hoc test for all graphs, ns nonsignificant.

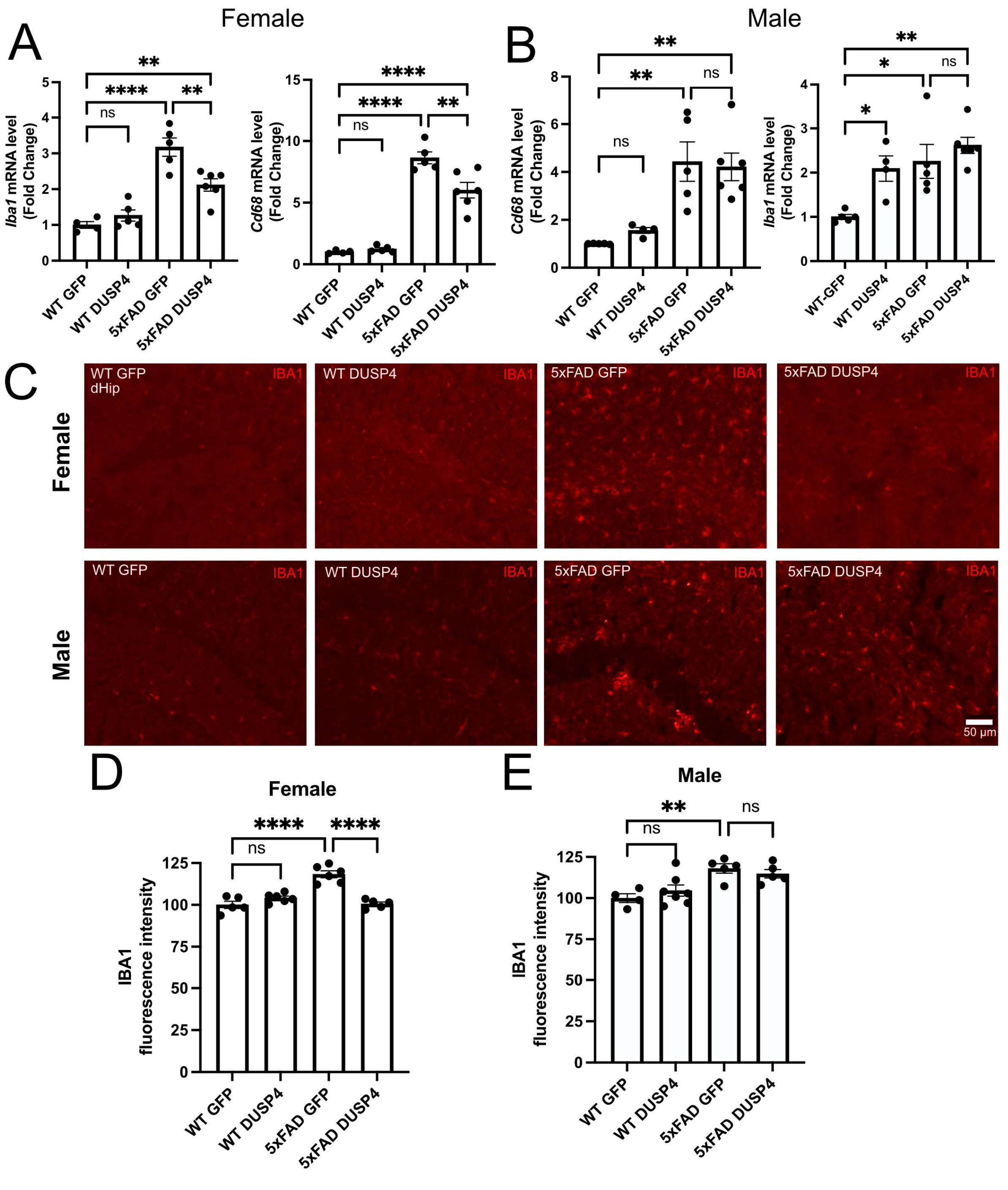

Figure S10. DUSP4 overexpression mitigated microglial activation in female 5xFAD mice, but not in male 5xFAD mice. (A) RT-qPCR results showed a significant decrease of Iba1 and Cd68

mRNAs in DUSP4-overexpressing female 5xFAD mice compared to GFP-overexpressing 5xFAD mice*, n = 4-6 mice/group.* (B) DUSP4-overexpressing male 5xFAD mice did not show any change in Iba1 and Cd68 mRNAs, n = 4-6 mice/group. (C) Representative images of microglial cells from female (top panel) and male (bottom panel) dorsal hippocampi labeled with anti-IBA1 (red). Scale bar = 50 µm. (D) Quantification of IBA1 fluorescence intensity from images in C (top panel) showed that DUSP4 overexpression decreased IBA1 levels in female 5xFAD, n = 5-6 mice/group. (E) Quantification of IBA1 fluorescence intensity from images in C (bottom panel) showed that DUSP4 overexpression did not change IBA1 levels in male 5xFAD mice, n = 4-6 mice/group. Statistical analyses were performed using a one-way ANOVA followed by a Tukey's post-hoc test for all graphs, *p<0.05, **p<0.001, ****p<0.0001; ns nonsignificant.
